# Risk of heavy metals accumulation in soil and wheat grains with waste water irrigation under different NPK levels in alkaline calcareous soil

**DOI:** 10.1371/journal.pone.0258724

**Published:** 2021-11-17

**Authors:** Maria Mussarat, Waqar Ahmad Jamal, Dost Muhammad, Manzoor Ahmad, Abida Saleem, Sowm Khan, Faiza Aman, Hamida Bibi, Wajid Ali Shah, Khadim Dawar, Noor ul Akbar, Ishaq Ahmad Mian, Muhammad Waheed, Irshad Ali, Afia Zia, Wasiullah Malik

**Affiliations:** 1 Department of Soil and Environmental Sciences, The University of Agriculture, Peshawar, Pakistan; 2 Department of Soil and Environmental Sciences, Amir Muhammad Khan Campus, The University of Agriculture, Peshawar, Pakistan; 3 Department of Agriculture, Bacha Khan University, Charsadda, Khyber Pakhtunkhwa, Pakistan; 4 Directorate General of Soil Water Conservation, Peshawar, Pakistan; 5 Department of Zoology, Kohat University of Science and Technology, Kohat, Pakistan; 6 Department of Agricultural Chemistry, The University of Agriculture, Peshawar, Pakistan; 7 Kohat University of Science and Technology, Kohat, Pakistan; Gifu University, JAPAN

## Abstract

A field study was conducted on the reuse of wastewater from Mardan city to evaluate its risk of contaminating soil and wheat grains at different NPK levels. Three irrigation sources i.e. waste water (WW), canal water (CW) and alternate waste + canal water (WW+CW) were applied to wheat (cv Atta Habib 2010) grown at 0, 50, 75 and 100% NPK levels of 120:90:60 kg N:P_2_O_5_:K_2_O ha^-1^ at Palatoo Research Farm, Amir Muhammad Khan Campus, Mardan during 2015.The results showed higher grain and biomass yields in WW irrigated plots as compared to CW at NPK levels up to 50% of recommending dose revealing supplementing nutrient requirements in deficient conditions. However, irrigation of WW at higher NPK levels especially at or beyond 75% of recommended dose tended to reduce the crop yield that could be associated with heavy metals toxicity and nutritional imbalances. The use of WW substantially increased AB-DTPA extractable Zn, Mn, Pb, Ni and Cd indicating a potential threat to soil contamination. Similarly, WW irrigated wheat had higher concentrations of these heavy metals as compared to CW which limits its use for production purposes without any remediation measures. The alternate use of CW and WW as revealed by its comparative lower contamination in soil and wheat than sole WW could be one of the possible solutions and may increase the time required for threshold soil contamination.

## Introduction

The urban wastewaters are frequently utilized for irrigation purposes in the outskirts of cities which are known to be rich in nutrients and reduce fertilizers. Which are the basic needs but also may pose potential threat to food and soil contamination depending on contaminants load, soil and climatic conditions and type of crops grown on such lands. With the increase in population, rapid industrialization and climate change demands for water increased several times. Like other developing countries and nations residing in fragile climates, Pakistan is not only facing the severe pressure of increasing population growth and demand for water but is also severely affected by climate change. Per capita water availability has decreased from 5260 m^3^ in 1951 to 1050 m^3^ by the year 2008 [1], placing Pakistan in the category of a high water stress country and now it is feared that this level might have reached about 900 m^3^ per capita.

Wastewater disposal is becoming a problem in developing countries as large quantities of municipal wastes and industrial effluents are being produced due to increased urbanization and industrialization. The situation is more aggravating in low-income provinces like Sind, Khyber Pakhtunkhwa and Baluchistan of the country where most of the agricultural lands are being continuously converted into Housing Scheme and irrigation systems into sewage channels. This untreated sewage water is applied to agricultural fields in nearby areas contaminating soils and crops grown in these areas. Due to poor literacy rate coupled with socio-economic conditions the farmers consider wastewater as a source of nutrition that results in high production. The long term application of wastewater to leguminous and cereal crops could result in a high growth rate, dry weight and increased yield [2, 3] but this must be on the cost of health safety and even the yield on a sustainable basis. Besides, its benefits these wastewaters may contain a substantial amount of heavy metals like lead (Pb), copper (Cu), Zinc (Zn), and Chromium (Cr) [4] and as such the long term use of these water could result in accumulation of heavy metals in the soil as well as could affect the uptake of applied fertilizers by making insoluble complexes in soil. In return, the major nutrients become unavailable to the plants and thus could result in decrease in yield [5] in poorly managed and fragile areas.

On one hand, the municipal wastewater, while containing high amounts of organic refuse may increase the yield. While on the other hand, this water could also interfere with the uptake of NPK that intern not only could affect plant biomass but also could stimulate the buildup of heavy metals in wheat grain [6] affecting human health. Pre-treatment of wastewater is always recommended before agricultural application but due to the high costs involved, in most places around the globe and more specifically in the developing countries, wastewater, irrespective of its origin is applied to the crops untreated. Therefore, ways are needed to decrease the mobility of metals, rendering their complication with applied fertilizers and thus decreasing their availability to the plant [7].

The wastewater applied to the experimental plot was received from the urban drain flowing in the farm. This drain receives untreated wastewater from household sewerage lines, industries, service stations, markets and subsurface drainage water from the porous pipes installed in agricultural lands of the area.

Keeping in view, the importance of wastewater in reducing the NPK demands wheat and its associated risk of increasing heavy metals concentrations in wheat production, the present study was conducted in field conditions at Platto Research Farm, Mardan using city wastewaters as a source of irrigation in comparison to canal water and alternate use of both sources.

## Methods and materials

### Field study

**Fig 1:**
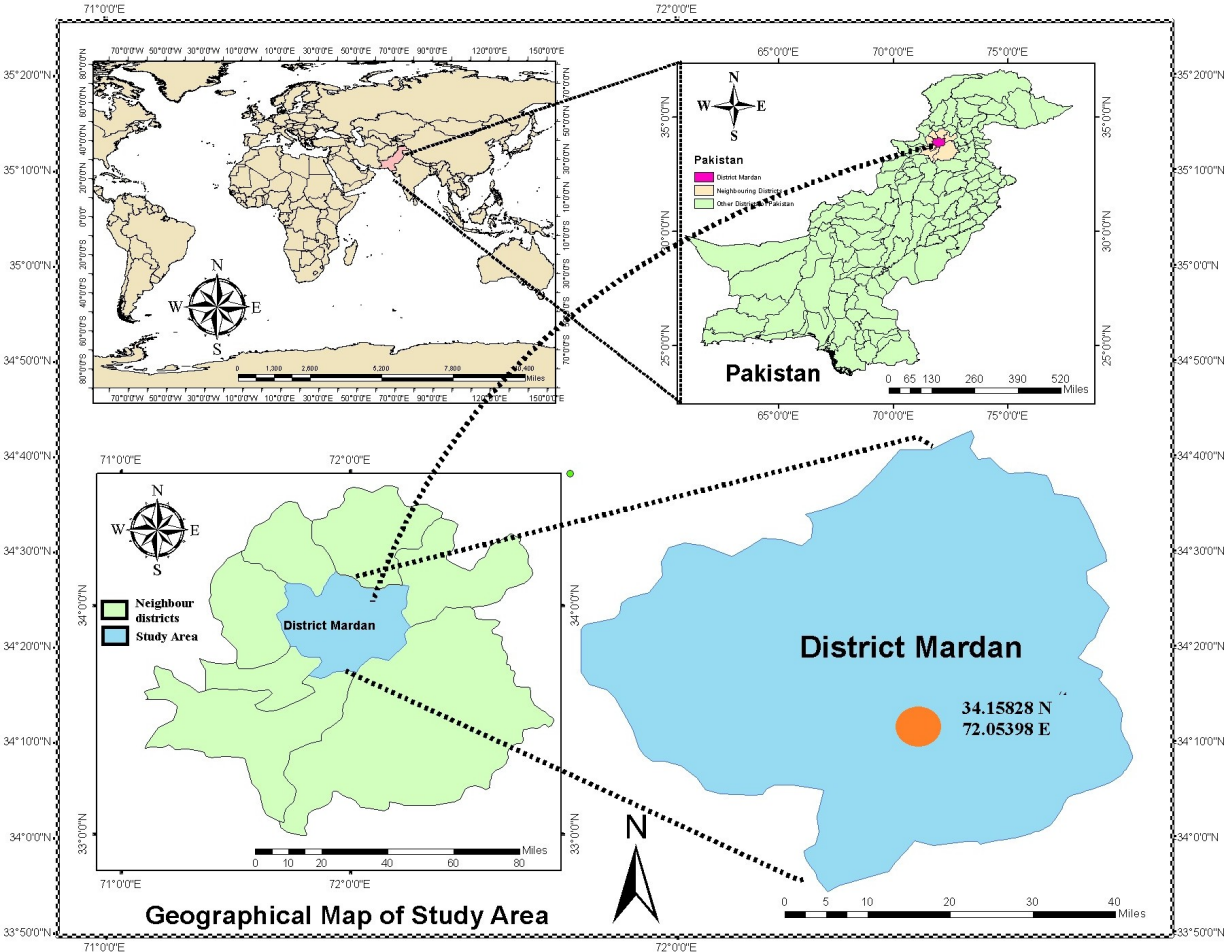
Map of the study area.

Three sources of water i.e. wastewater (WW), canal water (CW) and blending of both as alternate irrigation of WW and CW (WW + CW) were applied to wheat plots treated with 0, 50, 75 and 100% of recommended doses of NPK (120:90:60 kg N:P_2_O_5_:K_2_O ha^-1^) situated at Palatoo Research Farm, Amir Muhammad Khan Campus, Mardan the University of Agriculture Peshawar, Pakistan (Fig 1), in 2015. The map of the study area was designed by using ArcGIS software ver. 10.8. Fertilizers sources for NPK were Urea, Diammonium phosphate (DAP) and Muriate of Potash (MOP) respectively, which were incorporated in soil at the time of sowing whereas the wastewater was collected from the drain in the research farm receiving waste water from Mardan city and industrial effluents. The Canal water was received from lower Swat Canal system. The treated plot size was 3 x 4 m^2^ arranged in split-plot randomized completer block (RCB) design with three replications where the source of irrigation was placed in main plots and NPK levels in sub-plots. Data on crop growth, yield, and heavy metal concentration in soil and wheat grains were recorded per standard procedures. Heavy metals in soil and water were determined by the atomic absorption spectrophotometer (Shimadzu AA-6300), however, in soil, they were determined in AB-DTPA extractable solution [8] whereas in wheat grain, were determined in wet digested samples [9]. In soil analysis, 10 g soil was added with 20 mL AB-DTPA (Ammonium bicarbonate, diethylene triamine penta acetic acid) solution and shaken on a semi-orbital shaker for 30 minutes. The suspension was filtered through Whatman 42. For wheat grains analysis, a 0.5 g ground sample was added with 10 mL concentrated HNO_3_ and 4 mL HClO_4_ for 24 h and then heated at 350°C until the brownish colour of the suspension was disappeared. The volume was made up to 20 mL with the addition of distilled water and then filtered through Whatman 42. Standards were prepared in respective acidic and AB-DTPA solutions.

### Study site

The soil under study was silty soil with 90% silt and 7.4% clay. The pH was alkaline, which was checked by digital pH and EC meter with the value of 7.56 but not-saline having much lower soil EC of only 0.19 dS m^-1^. The soil was low in OM, N and P but adequate in soil K with values of 0.01% N, 2.7 mg P kg^-1^ and 115 mg kg^-1^ soil (Table 1). The analysis of wastewater shows that non-of heavy metal was beyond the permissible limit when compared with the standard values suggested by Food and Agriculture Organization (FAO) of the United Nations [10], and National Environmental Quality Standards (NEQS) [11] of Pakistan (Table 2). However, the EC value of this wastewater was higher qualifying as C3 water [12] suggesting careful management to avoid salinity in the soil in long term.

**Table 1:** Physico-chemical characteristics of experimental soil.

Properties	Values	Units
Sand	12.6	%
Silt	80	%
Clay	7.4	%
Textural class	Silt	-
pH_(1:5)_	7.64	-
EC_(1:5)_	0.19	dS m^-1^
SOM	0.94	%
N contents	0.014	%
P contents	2.7	mg kg^-1^
K content	115	mg kg^-1^

**Table 2:** pH, EC and heavy metal concentrations of waste water used in the study along with permissible limits for irrigation water.

Properties	Value±STD	NEQS [11]	FAO, 1985 [10]
pH_(1:5)_	7.77±0.20	6–9	6.5–8.4
EC_(1:5) dS m_^-1^	1.49±0.30	-	-
Cu (mg L^-1^)	0.057±0.08	1.0	0.2
Fe (mg L^-1^)	0.120±0.10	8.0	2.0
Mn (mg L^-1^)	0.020±0.02	1.5	2.0
Zn (mg L^-1^)	0.032±0.02	5.0	2–5
Ni (mg L^-1^)	0.028±0.01	1.0	0.02
Pb (mg L^-1^)	0.01±0.01	0.5	5.0
Cd (mg L^-1^)	0.02±0.01	0.1	5.0

### Laboratory analysis

Heavy metals in soil and water were determined by the atomic absorption spectrophotometer (Shimadzu AA-6300), however, in the soil they were determined in AB-DTPA extractable solution [8] whereas in wheat grain, were determined in wet digested samples [9]. In soil analysis, 10 g soil was added with 20 mL AB-DTPA (Ammonium bicarbonate, diethylene triamine penta acetic acid) solution and shaken on a semi-orbital shaker for 30 minutes. The suspension was filtered through Whatman 42. For wheat grains analysis, a 0.5 g ground sample was added with 10 mL conc. HNO3 and 4 mL HClO4 for 24 h and then heated at 350oC until the brownish colour of the suspension was disappeared. The volume was made upto 20 mL with the addition of distilled water and then filtered through Whatman 42. Standards were prepared in respective acidic and AB-DTPA solutions.

Soil samples were collected at a depth of 0–20 cm before sowing and after crop harvest of experimental plots. These samples were air-dried under shade conditions followed by sieving through a 2 mm sieve. The soil samples were then properly labelled and stored in plastic bags for further analysis. Heavy metals in soil and water were determined by the atomic absorption spectrophotometer, however, in soil they were determined in AB-DTPA extractable solution [8] whereas in wheat grain, were determined in wet digested samples [9].

### Statistical analysis

The data collected was analyzed statistically according to the procedure given by Jandel Scientific (1991) [13] using STATISTIX, 2000 package and Least Significant Difference (LSD) test was used for significant difference among the treatments [14].

## Results and discussions

### Grain yield

**Fig 2:**
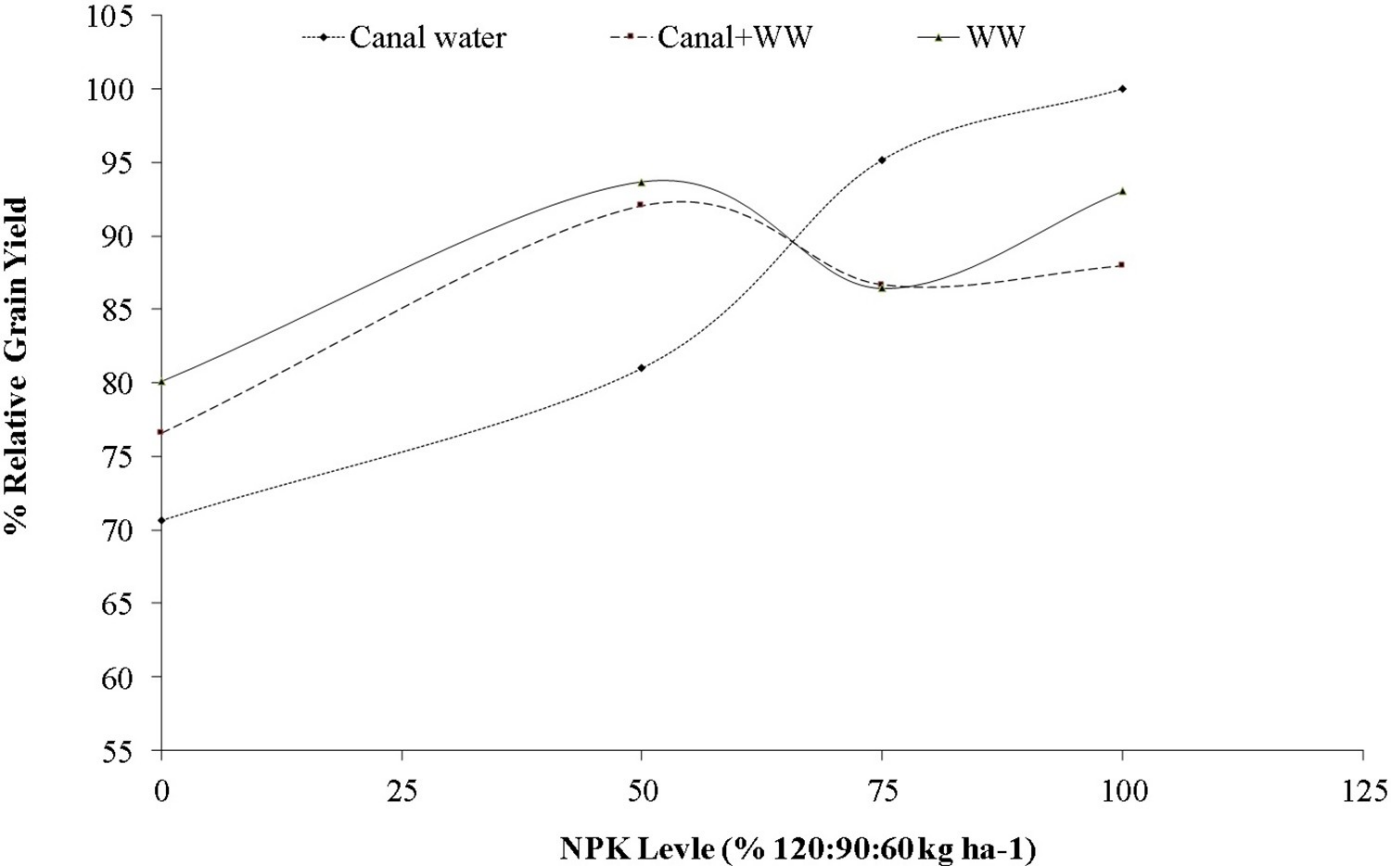
Relative percent of grain yield of wheat as affected by different NPK levels and irrigation source.

The result showed that the application of NPK and the interaction between NPK and irrigation sources significantly (p< 0.05) increased the grain yield of wheat. (Table 3). The maximum grain yield of 3111±136 kg ha^-1^ was recorded with the application of 100% recommended NPK in plots irrigated with CW followed by 75% NPK+ CW water and 50% NPK+WW with values of 2961±118 and 2914±58 kg ha^-1^, respectively. The non-significant difference in these values suggested saving in fertilizer requirements with the use of WW water irrigation. Wastewater (WW) irrigation had a higher grain yield of 2748±217 kg ha^-1^ as compared to CW or CW+WW when averaged across the soil-applied NPK levels. The use of WW increased the grain yield by 13.4% at the 0 NPK level and by 15.6 at 50% NPK levels when compared with CW. While a further increase in NPK levels tended to reduce its yield but in CW irrigation the grain yield increased linearly with an increase in NPK levels up to 100% NPK. The per cent relative yield (Fig 2) elaborated the same trend showing 80–100% of relative yield at 0–50% NPK levels whereas in the case of CW the relative yield was 70% at 0 NPK level which linearly raised to 100% at 100% of recommending NPK levels (120:90:60 kg N:P_2_O_5_:K_2_O ha^-1^). The decrease in yield above 50% of NPK level with WW could be attributed to nutritional imbalances.

**Table 3:** Wheat biomass and yield as influenced by NPK level and irrigation source.

Irrigation	NPK*	Biomass	Grain Yield
	--kg ha^-1^--	-------- kgha^-1^-------	
Canal water	0	4093±99 h	2198±68 h
Canal water	50	4905±264 g	2520±56 eg
Canal water	75	5931±341 ac	2961±118 ab
Canal water	100	6336±261 a	3111±136 a
Canal +wastewater	0	4218±300 h	2382±202 gh
Canal +wastewater	50	5518±473 ce	2863±115 bd
Canal +wastewater	75	5469±173 de	2696±161 cf
Canal +wastewater	100	5343±299 ef	2736±104 ce
Waste water	0	4941±142 fg	2493±165 fg
Waste water	50	5937±170 ab	2914±58 ac
Waste water	75	6104±110 ab	2689±132 df
Waste water	100	5764±208 bd	2894±189 ad
LSD (p<0.05)		412.37	221.58
Average across Irrigation source			
-	0	4417±433 c	2358±187 c
-	50	5454±532 b	2765±198 b
-	75	5835±347 a	2782±180 b
-	100	5814±486 a	2914±207 a
LSD (p<0.05)		238.08	127.9
Average across Fertilizer level			
Canal water	-	5316±943 b	2697±387
Canal +wastewater	-	5137±625 b	2669±225
Waste water	-	5687±487 a	2748±217
LSD (p<0.05)		206.18	NS

*100% NPK mean 120:90:60, 75% NPK mean 90:68:45 and 50% NPK mean 60:45:30.

The role of N, P and K in increasing the wheat yield is well established [15] and the present wastewater having appreciable amounts of NPK along with other micronutrients and organic material increased the wheat yields in our study which is not uncommon. Similar results were reported in another study [16] that wheat irrigated with wastewater produced taller plants, and higher grain yield than wheat grown with underground tube well water.

### Wheat biomass

**Fig 3:**
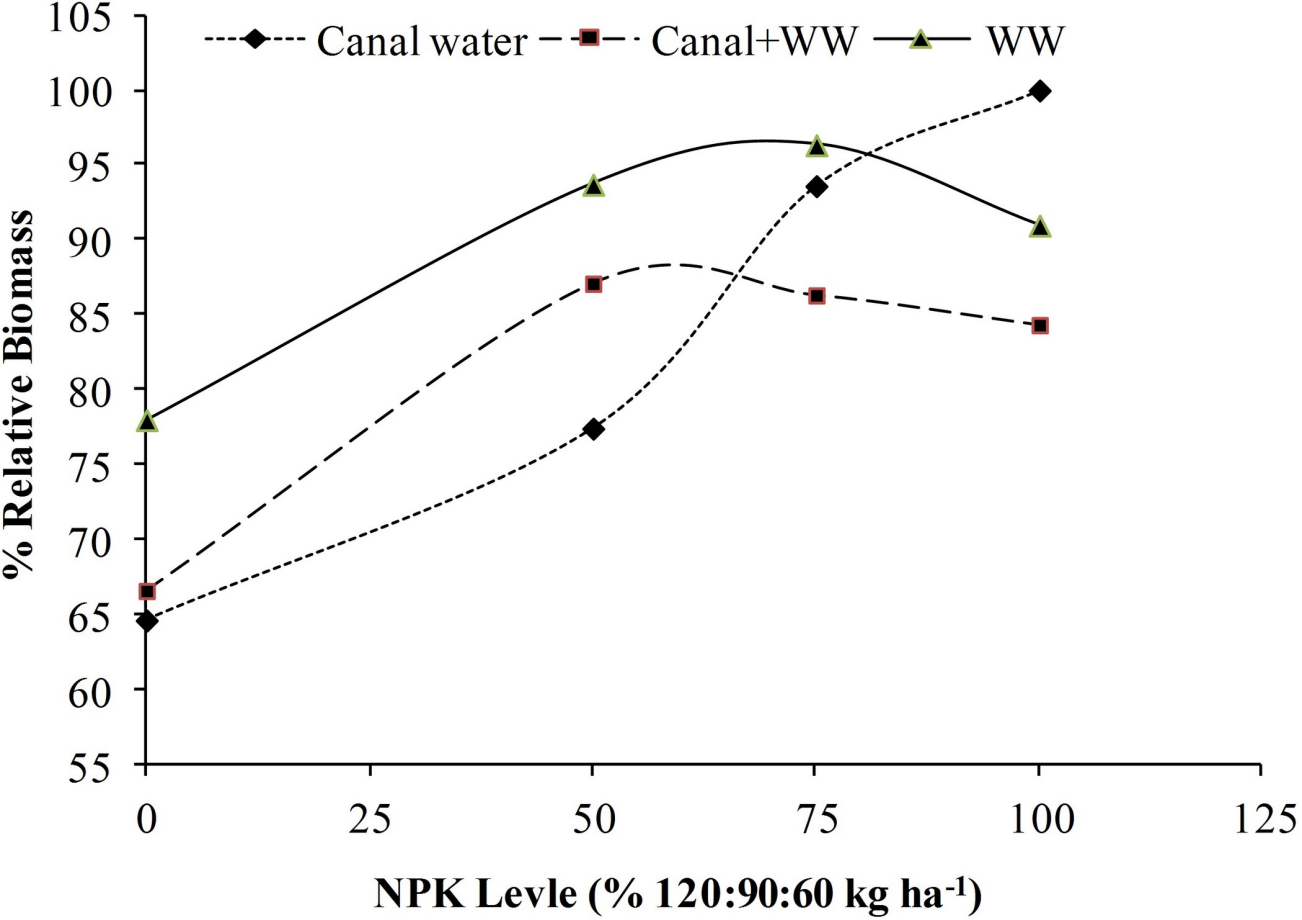
Per cent relative biomass of wheat as influenced by different NPK levels and irrigation source.

Like grain yield, the maximum biomass of 6336±261 kg ha^-1^ was also observed at 100% recommended NPK level irrigated with CW followed by 6104±110 kg ha^-1^ recorded at 75% NPK level+ WW (Table 4). The closely similar biomass values at 100% NPK+CW and 75% NPK+WW as well as at 75% NPK+CW and 50% NPK+WW indicated saving in fertilizer with the use of wastewater. When averaged across NPK levels, WW produced a significantly higher yield of 5687±487 kg ha^-1^ as compared to CW or CW+WW. Though on an average basis the biomass did not increase beyond 75% NPK levels in fact, this level depended on water irrigation sources. In the case of CW, the increase in NPK level linearly increased the crop biomass with each increment from 0 to 100% NPK level while in the case of WW or CW+WW this increase was not observed beyond 75% NPK even the differences between 75 and 50% NPK level were found non-significant. The same pattern was elaborated by (Fig 3) where 80 to 100% relative biomass was observed at 0 to 75% NPK +WW but in the case of CW, the relative biomass increased from 65% in 0 NPK level to 100% at 100% NPK level. Below 75% NPK the relative yield in WW was higher than the CW but after 75% the reverse was observed showing decreased at a higher NPK level with WW. The reduction could be attributed to imbalances in nutrients. The same reduction at a higher NPK levels with WW was also observed in the case of grain yield and plant height.

**Table 4:** Soil heavy metal (Mn, Fe, Zn and Cu) as influenced by NPK level and irrigation source.

Irrigation	NPK*	Mn	Fe	Zn	Cu
	-kg ha^-1^-	------------------------mg kg^-1^---------------------			
Canal water	0	3.13±0.34	1.11±0.14	0.86±0.20	2.94 ±0.70
Canal water	50	4.08±0.84	1.38±0.33	0.71±0.11	3.56±0.99
Canal water	75	3.89 ±0.16	1.76±0.92	0.94±0.23	3.33±1.05
Canal water	100	5.06 ±0.98	1.60±0.12	0.77±0.35	3.05±1.28
Canal+ waste water	0	4.18±0.63	1.92±0.73	0.74±0.17	3.61±2.00
Canal+ waste water	50	5.55±0.12	1.71±0.50	0.90±0.21	4.01±1.38
Canal+ waste water	75	6.81±0.83	1.73±0.77	1.04±0.45	3.61±1.23
Canal+ waste water	100	6.82±1.94	1.91±0.58	1.15±0.30	4.23±1.64
Waste water	0	5.580.69	1.99±0.55	1.25±0.14	3.43±1.80
Waste water	50	5.53±0.96	1.77±0.37	1.29±0.43	3.07±0.81
Waste water	75	7.74±1.50	2.01±1.41	1.38±0.18	3.68 ±0.14
Waste water	100	6.33±0.55	2.12±0.90	1.40±0.31	3.59±0.36
LSD (p<0.05)		NS	NS	NS	NS
Average across Irrigation source					
-	0	4.30±1.17 c	1.67±0.63	0.95±0.26	3.32±1.42
-	50	5.05±0.97 bc	1.62±0.39	0.97±0.31	3.54±1.02
-	75	6.15±1.94 a	1.83±0.94	1.12±0.34	3.54±0.83
-	100	6.071.37ab	1.88±0.59	1.11±0.39	3.62±1.17
LSD (p<0.05)		1.03	NS	NS	NS
Average across Fertilizer level					
Canal water	-	4.04± 0.92b	1.46±0.50	0.82±0.22 b	3.22±0.91
Canal +wastewater	-	5.84±1.48 a	1.82±0.57	0.96±0.30 b	3.87±1.38
Waste water	-	6.30±1.26 a	1.97±0.78	1.33±0.25 a	3.44 ±0.89
LSD (p<0.05)		0.89	NS	0.23	NS

*100% NPK (120:90:60), 75% NPK (90:68:45) and 50% NPK (60:45:30).

Nitrogen, phosphorous and potassium while playing key roles in many biochemical, enzymatic and metabolic activities as well as serve as structural components of many plant compounds can increase the plant growth and yield parameters. The WW containing nutrients like NPK and micronutrients can increase the yield if used for irrigation. It was confirmed by other researchers that the biomass of wheat increased with the application of wastewater [17]. Similar results were established in another study [18] who reported the maximum biomass of soya bean with the combined use of NPK and city waste compost.

### Postharvest soil AB-DTPA extractable Zn, Mn, Fe and Cu

Irrigation sources significantly affected postharvest soil AB-DTPA extractable Zn and Mn and NPK levels affected only Mn whereas Fe and Cu were neither affected by NPK levels nor by irrigation sources (Table 4). The application of 75% recommended dose of NPK had higher [Zn] with a value of 1.12±0.34 mg kg^-1^ as compared to 50 and100% recommended NPK levels but they were statistically non-significant. However, WW irrigation had a significantly higher [Zn] having a value of 1.33±0.25 mg kg^-1^ followed by CW+WW (0.96±0.30 mg kg^-1^) and CW (0.82±0.22 mg kg^-1^). Post-harvest soil Mn increased with the application of NPK doses but beyond 75% NPK doses no significant increase was observed (Table 4). The application of 75% recommended NPK had an Mn value of 6.15±1.94 mg kg^-1^ that was significantly higher than values of 5.05±0.97 and 4.30±1.17 mg kg^-1^ observed at 50% and 0% NPK levels when averaged across irrigation sources. Manganese concentration was also significantly affected by irrigation sources. The application of WW had a higher [Mn] value of 6.30±1.26 mg kg^-1^ as compared to CW+WW and CW.

Similarly, when averaged across irrigation sources, the highest Fe value of 1.88±0.59 mg kg^-1^ was recorded with the application of 100% recommended dose of NPK as compared to 75 and 50% NPK doses (Table 4). The maximum [Fe] with the value of 1.97±0.78 mg kg^-1^ was produced by WW irrigation as compared to CW+WW and CW but the result was non-significant. However, [Cu] showed non-significant (p< 0.05) differences for both the main effect of NPK level, irrigation sources and as well as for their interaction. As indicated by the averaged results of fertilizer level, Cu concentration gradually tended to increase with the increase in NPK level and wastewater irrigation but these changes with non-significant at p < 0.05 (Table 4).

These results were found similar to the finding of Dougherty and Hall (1995) [19] who reported that long term use of wastewater irrigation resulted in the accumulation Zn, Cd, Cu, Ni, Pb, Mn, Fe and Cr in soils. The same results were also indicated by Bedbabis *et al*., (2015) [18] who observed that wastewater irrigation increased soil organic matter, salts and soil heavy metals such as Mn, Zn and Fe content as compared to well water. Such results were also reported by other researchers that irrigation with wastewater increased the accumulation of K, Na, Fe, Mn, Zn, Cu and B in the soil as compared to canal water irrigated areas [20].

### Post-harvest soil AB-DTPA extractable Pb, Ni and Cd

Irrigation sources significantly affected the soil AB-DTPA extractable [Pb], [Ni] and [Cd] whereas the NPK levels affected only the soil [Pb] but the interaction of irrigation sources and NPK levels was not significant for any of these parameters (Table 5). These results revealed that the heavy metals Pb, Ni and Cd were mainly influenced by the application of wastewater.

**Table 5:** Soil heavy metal (Pb, Ni and Cu) as influenced by NPK level and irrigation source.

Irrigation	-- NPK--*	Pb	Ni	Cd
	---kg ha^-1^--	-------------------mg kg^-1^-------------------		
Canal water	0	0.13± 0.04	0.04± 0.01	0.09± 0.02
Canal water	50	0.15 ± 0.06	0.04± 0.02	0.09± 0.02
Canal water	75	0.12 ± 0.06	0.06± 0.046	0.07± 0.02
Canal water	100	0.17± 0.02	0.05± 0.03	0.08± 0.02
Canal +wastewater	0	0.19± 0.05	0.06± 0.042	0.11± 0.05
Canal +wastewater	50	0.26± 0.03	0.06± 0.02	0.11± 0.04
Canal+ wastewater	75	0.21± 0.02	0.08± 0.02	0.14± 0.01
Canal +wastewater	100	0.25± 0.06	0.08± 0.01	0.15± 0.03
Waste water	0	0.19± 0.02	0.08± 0.02	0.16± 0.07
Waste water	50	0.30 ± 0.01	0.09± 0.02	0.20± 0.06
Waste water	75	0.28± 0.01	0.09± 0.01	0.22± 0.06
Waste water	100	0.28± 0.05	0.09± 0.01	0.22± 0.07
LSD (p<0.05)		NS	NS	NS
Average across Irrigation source				
-	0	0.17± 0.05 b	0.06± 0.02	0.12± 0.026
-	50	0.23± 0.08 a	0.07± 0.03	0.14± 0.07
-	75	0.20± 0.08 ab	0.08± 0.014	0.14± 0.07
-	100	0.23± 0.06 a	0.07± 0.013	0.15± 0.07
LSD (p<0.05)		0.039	NS	NS
Average across Fertilizer level				
Canal water	-	0.14± 0.05 c	0.05± 0.03 b	0.08± 0.02 c
Canal +wastewater	-	0.23 ± 0.05b	0.07± 0.02 a	0.13± 0.04 b
Waste water	-	0.26± 0.05 a	0.09± 0.01 a	0.20± 0.06 a
LSD (p<0.05)		0.0335	0.0207	0.0393

* 100% NPK (120:90:60), 75% NPK (90:68:45) and 50% NPK (60:45:30).

The wastewater irrigation had higher postharvest soil AB-DTPA extractable [Pb], [Ni] and [Cd] concentrations with values of 0.26±0.03, 0.09± 0.02 and 0.20±0.06 mg kg-1, respectively which were about two times higher than the values observed in CW irrigated water. The CW+WW irrigated plots had less Pb, Ni and Cd as compared to WW but higher than CW showing that heavy metal contamination was proportionally increased with the number of irrigations with WW water. The increase in soil heavy metals with WW irrigation was also reported by Dougherty and Hall (1995) that long term use of wastewater irrigation could result in contamination of soil by Zn, Cd, Cu, Ni, Pb, Mn, Fe and Cr. The same result was observed by other investigators that irrigation with wastewater increases the accumulation of K, Na, Fe, Mn, Zn, Cu and B in the soil as compared to canal water irrigated areas [20].

The application of NPK levels, when averaged across the irrigation sources significantly increased the postharvest soil AB-DTPA ext. [Pb] and also tended to increase Ni and Cd on an average basis. However, these changes in all Pb, Cd and Ni in the present study were very minute and seemed to be due to making averages across the irrigation sources because none of the interaction was significant. The phosphatic fertilizer may contain minute amounts of heavy metals and can contaminate soil but such changes in the present highly buffered and strongly calcareous soils cannot be expected in such a short duration study.

Permissible limits of heavy metal in soils on a total weight basis range from 50–750 mg kg^-1^ soil for Cu, 150–750, 150–1400 for Zn, 50–300 for Pb and 1–20 for Cd. Though such speculation on AB-DTPA ext. weight basis is baseless but still, we can say that AB-DTPA ext. Cu, Fe, Zn, Mn, Cd, Pb and Ni increased with the application of wastewater. According to permissible limits of United Kingdome (UK), European Union (EU) and United States (US) as reported by Hassan et al. (2013) [21] and Pendias and Pendias (1986) [22] while the maximum permissible limit of the heavy metals was described by Merian (1991) [23] and Pendias and Pendias, 1986) [22] as shown in Table 6.

**Table 6:** Permissible levels of trace metals (mg kg^-1^) in soil from US, UK and EU irrigated with contaminated water and in agronomic crop (mg kg^-1^).

Heavy metal	Permissible levels of trace metals (mg kg^-1^) in soil			Maximum permissible limit [23] (Book)
	USA [21]	UK [21]	EU [21]	
1Cu	750	135	50–140	73.30
Fe	-	-	-	-
Mn	-	-	-	500
Zn	1400	300	150–300	99.40
Ni	210	75	30–75	67.90
Pb	150	300	50–300	0.30
Cd	20	3	1–3	0.20
As	-	-	-	0.43

The AB-DTPA ext values in WW irrigated plots were 6.3±0.55 mg kg^-1^ for Mn, 1.92±0.73 for Fe, 1.33±0.25 for Zn and 3.44±0.89 for Cu (Table 4) whereas the Cd, Ni and Pb were 0.20±0.06, 0.09±0.01 and 0.26±0.05 mg kg^-1^ soil respectively (Table 5). Hence the Cd toxicity in the soil could be expected which was close to lower from the permissible limit of Cd on a total weight basis.

### Wheat grain [Mn], [Fe], [Zn] and [Cu] concentrations

The analysis of the data showed that irrigation sources significantly (p< 0.05) affected wheat grain [Mn], [Fe], [Zn] and [Cu] over control whereas the NPK levels and the interaction between NPK and Irrigation source were not significant (Table 7).

**Table 7:** Grain heavy metals (mg kg^1^) as affected by different NPK level and irrigation source.

Irrigation	NPK	Mn	Fe	Zn	Cu
	-kg ha^-1^-	-------------mg kg ^-1^---------------			
Canal water	0	288.0± 39.5	148.8± 20.6	24.2±3.4	31.2±5.9
Canal water	50	290.4± 61.9	149.1± 4.5	27.8±4.5	36.6±9.9
Canal water	75	312.7± 10.4	158.5±22.8	28.9±3.9	34.7±5.8
Canal water	100	282.4± 39.3	150.9 ±37.7	23.1±3.0	38.8±3.2
Canal+ waste water	0	331.3± 23.8	197.7 ± 37.8	32.8±2.1	49.7±4.9
Canal+ waste water	50	345.6± 14.9	202.7± 39.4	34.5±2.8	56.2±7.9
Canal+ waste water	75	329.9± 52.8	221.6± 19.9	34.0±5.2	60.1±4.2
Canal+ waste water	100	361.6± 29.3	235.8 ±8.3	36.7±5.2	58.9±3.0
Waste water	0	361.8± 41.1	286.5± 62.3	42.5±5.1	78.9±8.0
Waste water	50	359.8± 28.4	293.6± 12.7	44.6±1.6	74.8±5.3
Waste water	75	373.9± 24.9	275.5±25.8	44.3±3.7	76.5±9.8
Waste water	100	380.2± 30.6	270.3± 28.0	42.2±7.2	81.9±2.3
LSD (p<0.05)		NS	NS	NS	NS
Average across Irrigation source					
-	0	327.0± 44.5	211.0±71.3	33.2±8.6	53.2± 21.6
-	50	331.9± 47.1	215.1±66.6	35.6±7.8	55.9±17.9
-	75	338.8± 40.3	218.5±54.5	35.7±7.7	57.1±19.6
-	100	341.4± 53.5	219.0±58.3	34.0±9.7	59.7±18.8
LSD (p<0.05)		NS	NS	NS	NS
Average across Fertilizer level					
Canal water	-	293.4± 37.8 b	151.8±21.2 c	26.0±4.1 c	35.3±6.4 c
Canal +wastewater	-	342.1± 31.4 a	214.5±29.6 b	34.5±3.7 b	56.1±6.1 b
Waste water	-	368.9± 28.6 a	281.5±32.9 a	43.4±4.3 a	78.0±7.3 a
LSD (p<0.05)		26.904	23.412	3.4943	5.8031

*100% NPK (120:90:60), 75% NPK (90:68:45) and 50% NPK (60:45:30).

When averaged across irrigation sources, [Mn] in wheat grain was higher in WW irrigation as 368.9± 28.6 mg kg^-1^ followed by CW+WW and the least as 293±37.8 mg kg^-1^ were observed in CW. Mn values were still within the permissible limits as suggested by some researchers [23]. Data regarding [Fe] in grain showed 85.4% than control with WW irrigation when values were averaged across the NPK level. However, this higher value of 281.5±32.9 mg kg^-1^ in WW plots was still within safe limits (WHO) [24]. Comparing the average values across irrigation sources, [Zn] in wheat grain was also higher in WW irrigated plots with the value of 43.4±4.3 mg kg^-1^ followed by CW+WW while the lowest 26.0±4.1 mg kg^-1^ were recorded in CW irrigation. WW irrigation containing Zn would have caused an increased in soil and plant Zn, however, these values are still below the safe limits and the scientists are intending to increase the wheat grain Zn up to 50 mg kg^-1^ to reduce the Zn deficiency in consumers. Similar to Mn, Fe, Zn the wheat grain [Cu] also increased with WW water and as such induced the highest value of 78.0±7.3 mg kg^-1^ among the irrigation sources. The mixed irrigation of WW and CW caused 56.1±6.1 mg Cu ha^-1^ and CW as 35.3 ±6.4 mg Cu kg^-1^. All the values were, however, lower than the safe limits [23]. The application of 100% of recommended dose increased [Cu] in grains to 59.7±18.8 mg kg^-1^ as compared to 53.2±21.6 mg kg^-1^ observed in 0% NPK however this difference was statistically not significant.

These results suggested that WW containing heavy metals would have increased the Cru, Fe, Zn and Mn in grains. Kisku *et al*. (2000) [25] observed increases in plant uptake of Cd, Cu, Ni and Pb when irrigated with industrial effluent. Arora *et al*. (2008) [26] also reported that wastewater irrigation increased concentrations of heavy metals in radish, spinach, turnip, brinjal, cauliflower and carrot as compared to canal water irrigation. Similarly, the increase in grain Zn with WW was reported by Togay *et al*. (2008) [27] who observed that the application of wastewater significantly increased Zn concentration in seed and Cu and Zn in the shoot of the plant. These results were also similar to the finding of Singh and Agrawal (2010) [28] who concluded that heavy metal concentration in rice increased with wastewater irrigation.

### Wheat grain [Pb], [Ni] and [Cd] concentrations

The analysis of the data showed that irrigation sources significantly (p< 0.05) affected wheat grain [Pb], [Ni] and [Cd] over control whereas the NPK level significantly influenced only grain [Pb] while effects on grain [Ni] and [Cd] were not significant (Table 8). When averaged across irrigation sources, WW irrigated plot significantly increased [Pb] in wheat grain with the value of 2.29±0.41 mg kg^-1^ as compared to canal plus wastewater and canal water. As indicated by the averaged result of fertilizer levels, the NPK fertilizer significantly increased the grain [Pb] and the maximum mean value of 1.86±0.55 mg kg^-1^ was observed by the 100% NPK dose. The fertilizer especially the phosphatic fertilizer may contain traces of Pb that would have increased the grain [Pb]. The plots receiving WW water had grain Pb values from 1.92±0.52 to 2.53±0.14 suggesting that application of WW at any NPK level may pose threats of Pb contamination in wheat grains. The permissible limit of Pb in plant is 2.0 mg kg^-1^ [24] and 0.30 [23]. The Ni values also increased with WW irrigation and significantly higher 1.53±0.31 mg Ni kg^-1^ were observed in these plots as compared to 1.37±0.25 g ha^-1^ in CW+WW and 0.91±0.20 mg kg^-1^ in CW irrigated plot when averaged across the NPK levels. However, the Ni values on average were in the safe limit [23].

**Table 8:** Grain heavy metal (Pb, Ni and Cu) as influenced by NPK levels and irrigation source.

Irrigation	NPK*	Pb	Ni	Cd
	-kg ha^-1^-	-----------------mg kg^-1^----------------		
Canal water	0	0.97±0.08	0.88±0.10	0.36±0.10
Canal water	50	1.27±0.04	0.95±0.33	0.29±0.14
Canal water	75	1.18±0.12	0.96±0.12	0.48±0.04
Canal water	100	1.32±0.08	0.85±0.27	0.39±0.24
Canal +wastewater	0	1.54±0.16	1.27±0.19	0.38±0.16
Canal +wastewater	50	1.64±0.20	1.38±0.19	0.53±0.06
Canal+ wastewater	75	1.67±0.20	1.42±0.24	0.52±0.05
Canal +wastewater	100	1.75±0.24	1.39±0.43	0.56 ±0.09
Waste water	0	1.92±0.52	1.67±0.28	1.20±0.08
Waste water	50	2.22±0.38	1.33±0.23	1.07±0.20
Waste water	75	2.51±0.32	1.57±0.38	1.13±0.14
Waste water	100	2.53±0.14	1.55±0.40	1.10±0.32
LSD (p<0.05)		NS	NS	NS
Average across Irrigation source				
-	0	1.47±0.50 b	1.27±0.38	0.65±0.43
-	50	1.71±0.47 ab	1.22±0.30	0.63±0.37
-	75	1.780.62± a	1.31±0.36	0.71±0.33
-	100	1.86±0.55 a	1.26±0.45	0.68±0.38
LSD (p<0.05)		0.24	NS	NS
Average across Fertilizer level				
Canal water	-	1.18±0.16 c	0.91±0.20 b	0.38±0.14 b
Canal +wastewater	-	1.65±0.19 b	1.37±0.25 a	0.49±0.11 b
Waste water	-	2.29±0.41 a	1.53±0.31 a	1.13±0.18 a
LSD (p<0.05)		0.21	0.24	0.12

*100% NPK (120:90:60), 75% NPK (90:68:45) and 50% NPK (60:45:30).

The wheat grain [Cd] also increased with WW irrigation and produced 1.13±0.18 mg Cd kg^-1^ when averaged across the NPK levels. The WW irrigated plots had 1.20±0.08 mg Cd kg^-1^ at 0 NPK level to 1.13±0.14 mg Cd kg^-1^ at 75% NPK level which was higher about 100% than plots irrigated with CW at the same NPK level corroborating that wastewater containing Cd would have increased the grain [Cd]. The permissible limit of Cd in the plant is 0.20 mg kg^-1^ of the plant body [23].

The WW water containing Pb, Ni and Cd would have increased these elements in soil resulting in their high accumulation in wheat grains. It was observed in another study that Cd accumulation occurred in plants irrigated with wastewater in South Tehran [29]. Mapanda et al. (2005) [30] also reported significant increases in concentrations of Cu, Zn, Cd, Ni, Cr and Pb in the topsoil of sites irrigated with wastewater increased as compared with the control soils and subsoil. Similarly, Khan et al. (2008) [31] reported higher concentrations of Cd, Ni, Pb, Cu and Cr in the plant irrigated with wastewater.

## Conclusions

The relatively higher grain yield of WW irrigated plots at 50% applied than 75% of NPK level in CW irrigated plots suggested a decrease in NPK requirement with wastewater irrigation. However, it also caused significant increases in AB-DTPA ext. Zn, Mn, Cd, Ni and Pb posing a potential threat to their accumulation over time. It was reflected in the wheat grains which had about 2 folds higher heavy metals over CW irrigated plots. It was concluded that though the wastewater could reduce the NPK requirements but still due to higher accumulations and concentrations of heavy metals in wheat grain their use cannot be recommended without proper monitoring and management strategies.
